# Differential Effects of Alpha-Particle Radiation and X-Irradiation on Genes Associated with Apoptosis

**DOI:** 10.1155/2011/679806

**Published:** 2011-06-09

**Authors:** Vinita Chauhan, Matthew Howland, Jeremy Chen, Barbara Kutzner, Ruth C. Wilkins

**Affiliations:** Consumer and Clinical Radiation Protection Bureau, Healthy Environments and Consumer Safety Branch, Health Canada, 775 Brookfield Road, PL 6303B, Ottawa, ON, Canada K1A 0K9

## Abstract

This study examined differential effects of alpha-(**α**-) particle radiation and X-rays on apoptosis and associated changes in gene expression. Human monocytic cells were exposed to **α**-particle radiation and X-rays from 0 to 1.5 Gy. Four days postexposure, cell death was measured by flow cytometry and 84 genes related to apoptosis were analyzed using real-time PCR. On average, 33% of the cells were apoptotic at 1.5 Gy of **α**-particle radiation. Transcript profiling showed statistical expression of 15 genes at all three doses tested. Cells exposed to X-rays were <5% apoptotic at *~*1.5 Gy and induced less than a 2-fold expression in 6 apoptotic genes at the higher doses of radiation. Among these 6 genes, Fas and TNF-**α** were common to the **α**-irradiated cells. This data suggests that **α**-particle radiation initiates cell death by TNF-**α** and Fas activation and through intermediate signalling mediators that are distinct from X-irradiated cells.

## 1. Introduction

Over the preceding decade, alpha-(*α*-) particle radiation has become a prominent public health concern predominately due to its presence in the occupational environment (i.e., medical imaging and cancer therapy) [1] and residential environment (i.e., radon (^222^Rn) gas). Particular concern has been raised over residential ^222^Rn exposures, as recent studies have shown a positive correlation between the development of lung cancer and exposure to ^222^Rn gas [2, 3]. Due to its high damaging power, it has been shown that the rate of repair to DNA-damaged cells after *α*-exposure is much slower compared to a low linear energy transfer (LET) irradiation such as X-rays [4]. This is because alpha-particles induce multiple ionizations within the DNA structure and in adjacent molecules [5] resulting in severe locally damaged sites that the cell is less likely to repair [6]. It has been shown that chromosome damage from *α*-particles is about 20 times greater than that caused by an equivalent dose of X-rays [7]. Both *in vitro* and animal-based studies have provided evidence to support such adverse cytogenetic effects. Studies conducted in mice have shown increased chromosomal instability in haemopoietic cells following exposure to *α*-particle radiation [8]. Recently, Li et al. have shown that a single dose of *α*-particle radiation could induce malignant transformation in benign prostate epithelial cells [9]. Despite the multitude of studies showing cytogenetic effects associated with *α*-particle exposure, clear mechanisms leading to *α*-particle-radiation-induced carcinogenesis have not been clearly defined, particularly at the transcript level. There is a possibility that these effects may be mediated through the modulation of expression levels in genes related to DNA damage and apoptosis. 

It is well known that radiation-damaged cells are prone to undergo apoptosis and failure to do so may lead to carcinogenesis [10]. To date, the apoptotic pathways involved in the different types of ionizing radiation (i.e., X-rays, gamma, and alpha) have not been clearly defined or compared [11]. Apoptosis, or programmed cell death, is a critical component of homeostasis in organs and tissues [12]. Different gene families regulate the characteristic sequence of events of apoptotic cell death which are essential to the development and function of an organism. During apoptosis, cells that are no longer needed are systemically eliminated, thereby preventing the development of an inflammatory response, which is often associated with necrotic cell death [13]. Some cell types are more sensitive to undergoing apoptosis and the signaling pathways employed by these different cell types to induce apoptosis may vary accordingly. Radiation-induced apoptotic signaling can be initiated in different cellular compartments, including the nucleus, cytosolic elements, and plasma membrane [11]. Simply described, the process of apoptosis is mediated by a family of caspases, which are inactive enzymes that become proteolytically active upon initiation of a stress signal. Two pathways have been defined that lead to caspase activation: the extrinsic pathway is initiated by ligation of transmembrane death receptors (CD95, TNF receptor, and TRAIL receptor) to activate membrane-proximal caspases which in turn cleave effector caspases (caspase 3 and caspase 7). The intrinsic pathway requires the disruption of mitochondrial membrane and the release of mitochondrial proteins that induce activation of caspase 9 and 5, thereby initiating the apoptotic pathway [14, 15]. There is considerable crosstalk between the extrinsic and intrinsic pathways. For radiation-exposed cells, caspase activation has been shown to be central to the apoptotic process. 

The aim of this paper was to examine the apoptotic response invoked by high-LET *α*-particle radiation and compare this response to a low-LET radiation type (i.e., X-ray) at equivalent dose rates. The purpose was to assess transcript modulations associated with these different radiation types in order to gain a comparative understanding of the mechanisms of radiation-induced apoptosis and biological effects. To date, the apoptotic response and associated gene modulations have not been studied in-depth following exposure of cells to *α*-particle radiation. The dysregulation of apoptotic gene expression has also been proposed to create a platform that is necessary and sufficient for tumor formation [16]. These studies were conducted in a monocytic cell line, as these cells are the first line of defense against foreign matter in the body and are known to produce strong inflammatory responses in the presence of stressors. The expression of 84 key genes involved in apoptosis was assessed after exposure to *α*-particle radiation or X-radiation using quantitative real-time PCR (qRTPCR). The array included the TNF ligands and their receptors; members of the BCL2 family, caspase, IAP, TRAF, CARD, death domain, death effector domain, and CIDE families; genes involved in the p53 and ATM pathways. 

In addition, apoptosis was also detected using a multi-caspase assay and annexin V conjugate staining. Caspase enzymes play a central role in the apoptotic cell death process as they form a family of enzymes that initiate and modulate the apoptotic cascade resulting in cell death through proteolysis of specific substrates [17]. The multi-caspase detection assay determines the expression of these enzymes, which can be interpreted as activation of caspase effectors causing apoptotic cell death. Annexin V is a protein which binds to phosphatidylserine (PS). Although PS is usually found on the inside of the cell membrane, during the apoptotic process there is an inversion of the leaflets, causing PS to be displayed on the outside of the cell. This allows annexin V to bind to the cell and serve as a marker of the apoptotic process [18].

## 2. Materials and Methods

### 2.1. Cell Culture

A human monocytic cell line (THP-1) cultured from the blood of a male with acute monocytic leukemia was obtained from American Type Culture Collection (ATCC, Manassas, Va, USA). THP-1 cells were maintained in a humidified incubator (37°C, 5% CO_2_/95% air) in 75 cm^2^ tissue culture flasks (Costar, Cambridge, Ma, USA). The cells were grown to confluence for 2-3 days in Royal Park Medical Institute-1640 (RPMI-1640) (Invitrogen Canada, Burlington, ON, Canada) medium, containing 10% fetal bovine serum (FBS) (Sigma-Aldrich Canada, Oakville, ON, Canada). A total of 1.0 × 10^6^ cells were seeded into 5 mL of culture media containing 100 units/mL of penicillin and 100 *μ*g/mL of streptomycin (Invitrogen Canada Inc.). The cells were exposed to *α*-particle radiation at doses ranging from 0.0 (control) to 1.5 Gy, using ^241^Americium-(^241^Am) electroplated discs (Eckert and Ziegler Isotope Products Ltd, Valencia, Calif, USA) having an activity level of 66.0 kBq ± 3% (dose rate of 0.98 ± 0.01 Gy/h, LET of 127.4 ± 0.4 keV/*μ*m). The absorbed dose of *α*-radiation to which cells were exposed was calculated using the GEANT4 v.9.1 Monte Carlo toolkit [19]. For the *α*-particle exposures, cells were cultured in thin Mylar-based plastic dishes (MD) (Chemplex Industries, Palm City, Fla, USA), which allowed the penetration of the *α*-particles. Cells destined for X-radiation were irradiated using the X-RAD 320 X-ray irradiation system at a dose rate of 0.98 ± 0.05 Gy/h, 120 keV (Precision X-ray, Inc.).

### 2.2. RNA Extraction

Four days following exposure to *α*-particle, X-ray radiation, or negative control conditions, 5 mL of cell culture were transferred to 15 mL Falcon centrifuge tubes (Invitrogen, Canada) and centrifuged at 200 ×g for 5 min to pellet the cells. The supernatant was decanted and stored at −80°C until further analysis. The pelleted cells were resuspended in 350 *μ*L of Buffer RLT containing 1%  *β*-Mercaptoethanol (Qiagen's RNeasy Mini kit; Qiagen Inc, Mississauga, ON) then frozen at −80°C until processed. 

Frozen THP-1 cells were thawed on ice and mixed well by pipetting. The lysate was transferred directly onto a QIAshredder spin column (Qiagen Inc), placed in a 2 mL collection tube, and centrifuged for 2 min at ~12,000 g. A volume of 350 *μ*L of 70% ethanol was added. Total RNA was then extracted using the RNeasy Mini kit according to the manufacturer's instructions (Qiagen Inc), with the addition of Qiagen's On-Column RNase-free DNase (Qiagen Inc) to eliminate any remaining DNA contamination. All total RNA sample concentrations and RNA quality were determined using both an Agilent 2100 Bioanalyzer and RNA Nanochips (Agilent Technologies Canada Inc., Mississauga, ON) and spectrophotometrically using an Ultrospec 2100 (Fisher Scientific) (OD ratio of A260 : A280). All extracted RNA samples were determined to be of good quality (RNA Integrity Number = 10) with minimal degradation and stored at −80°C until further analysis.

### 2.3. qRTPCR Analysis

Total RNA (1 *μ*g) isolated from THP-1 cells was reverse transcribed into complementary DNA using the RT^2^ First Strand Kit (SuperArray Bioscience Corp., Frederick, Md, USA). Gene profiling was done as described by the manufacturer using the RT^2^ profiler PCR human apoptosis pathway array (Table 1). The relative expression of each gene was determined by using the comparative threshold (Ct) method [20].

### 2.4. Annexin V Assay

Four days postexposure, cell cultures were collected, transferred to 5 mL tubes (BD), and centrifuged at 200 ×g for 5 min to pellet cells. The cells were then washed in 1 mL PBS and resuspended in 100 *μ*L of 1x Annexin binding buffer (10 mM HEPES, 140 mM NaCl and 2.5 mM CaCl_2_, pH 7.4) (Invitrogen). Five microliters of annexin V FITC (Invitrogen) and 0.25 *μ*L of 50 *μ*g/mL propidium iodide (PI) (Millipore, Billerica, Mass, USA) were added to each sample and incubated for 15 min at room temperature in the dark. After incubation, 400 *μ*L of 1x binding buffer was added to each sample and analyzed by flow cytometry. For flow cytometry analysis, data acquisition was set to analyze 2.0 × 10^4^ cells from the whole cell population as identified by a forward scatter (FSC) versus side scatter (SSC) dot plot. All debris under the FSC and SSC thresholds was excluded from the analysis. Mid-apoptotic cells were identified as those having a positive annexin V signal and no PI signal, while late apoptotic cells were positive for both annexin V and PI. All samples were analyzed on a BD FACSCalibur flow cytometer (BD Biosciences, San Jose, Calif, USA).

### 2.5. Multicaspase Detection Assay

Four days postexposure, 5000 cells were placed in a 1 mL tube and washed with apoptosis wash buffers provided in the Guava Caspase kits (Millipore). These aliquots were further processed according to the manufacturer's instructions (Millipore). Data was acquired on a Guava PCA flow cytometer (Millipore). Data acquisition was set to examine 2000 cells per sample. Threshold cut-offs and gating were established using a negative and positive control. The positive control comprised cells that were treated for 24 hr with 10 *μ*M of camptothecin. Two populations of cells were identified: mid-apoptotic cell populations were caspase reagent^+^/7-ADD^−^ and late apoptotic cell populations are caspase reagent^+^/7-ADD^+^. The caspase reagent is an inhibitor consisting of a peptide specific for each caspase active site, conjugated to a carboxyfluorescein fluorchrome, as well as a fluoromethyl ketone group which covalently links the inhibitor to the activated caspase. Once inside the cell, the caspase inhibitor binds covalently to the caspases that have been activated. The resulting fluorescent signal in the cell is proportional to the number of active caspase enzymes that are present in the cell when the reagent was added. 7-AAD is an indicator of membrane structural integrity, as it is excluded form live healthy cells to mid-apoptotic cells, but permeates cells at later stages of apoptotic death.

### 2.6. Pentoxifylline Suppression of TNF-*α* Expression

THP-1 cells were seeded into 5 mL of complete media containing 100 mM of inhibitor pentoxifylline (PTX) (Sigma-Aldrich, Canada). Serial dilutions of PTX at concentrations ranging from 1 mM to 1000 mM were added to culture media in order to determine optimal concentrations of the inhibitor that were not cytotoxic to the cells. A concentration of 100 mM PTX was established as optimal which was non-toxic to the cells and also induced a biological response. The cells were exposed to *α*-particle or X-ray radiation at equivalent doses. Four days postexposure, cells were collected and analyzed using the multicaspase detection assay as per manufacturer's instructions (Millipore, Billerica, Mass, USA).

### 2.7. Statistical Analysis

For the multicaspase and annexin data sets, statistical significance was determined using a repeated measure design ANOVA with a Dunnett multiple comparison post hoc test using GraphPad InStat version 3.00 for Windows 95 (San Diego, Calif, USA, http://www.graphpad.com/). This program was also used to test for significant linear trends within data-sets. Data sets were based on *n* = 3 or *n* = 6 biological replicates. Analysis of qRTPCR expression profiles and statistical analysis of data were performed using the superarray biosciences web portal for data analysis of their products. (SABiosciences http://www.sabiosciences.com/pcr/arrayanalysis.php/).

## 3. Results

### 3.1. Cellular Viability

**Figure 1:**
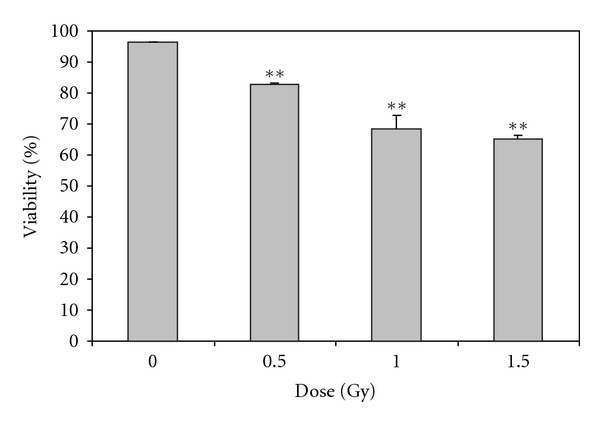
The percentage of viable cells in total cell population as a function of dose, 4 days after exposure to *α*-radiation as indicated by the multicaspase assay. Results are based on *n* = 3 biological replicates. **indicates statistically significant differences compared to the nonirradiated control (*P* ≤ .01). Figure is plotted as means ± SEM.

To assess the overall integrity of the cells, the viability and cell number were measured immediately prior to irradiation and 4 days following *α*-particle irradiation. Prior to irradiation, the cells remained 95–99% viable and no statistically significant differences in cell number were observed between groups (data not shown). At four days irradiation, statistically significant effects on cell viability were evident. Low doses of *α*-particle radiation caused significant decreases in cell viability (*P* ≤ .05) (Figure 1). At the highest dose of radiation (1.5 Gy), ~65% of the cells remained viable relative to the control treatment group (Figure 1).

### 3.2. Multicaspase Assay

**Figure 2:**
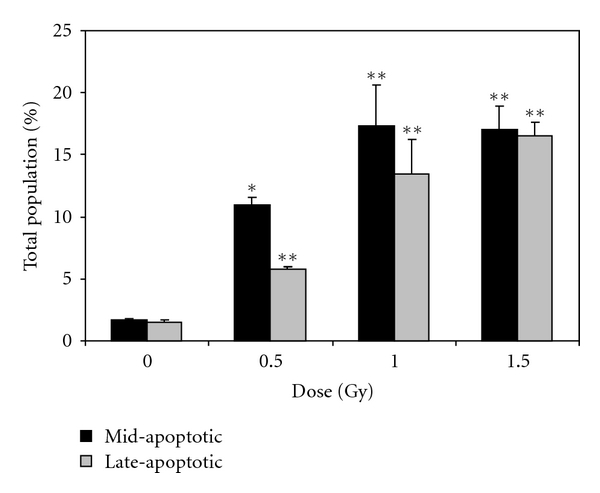
Percentage of mid- and late-apoptotic cells as a function of dose, 4 days after exposure to *α*-radiation as indicated by the multicaspase assay. Results are based on *n* = 3 biological replicates. *indicates statistically significant differences compared to the nonirradiated control (*P* ≤ .05). **indicates statistically significant differences compared to the nonirradiated control (*P* ≤ .01). Figure is plotted as means ± SEM.

The number of active caspase enzymes was assessed using a multicaspase detection assay. Analysis of caspase activity postexposure showed statistically significant increases in the percentage of cells with elevated caspase enzyme activity at all doses (0.5, 1.0, and 1.5 Gy) tested (Figure 2). At the lowest dose of irradiation, a greater percentage of cells were in the “mid-apoptotic stage” as indicated by the population of cells that were caspase reagent^+^/7-ADD^−^, while at the higher dose of *α*-particle radiation, equal numbers of cells were in “late-apoptotic phase” and “mid-apoptotic stage” as indicated by the population of cells that were caspase reagent^+^/7-ADD^+^. Overall, a linear dose-response curve was obtained for cells in late-apoptosis with approximately 17% of the cell population exhibiting advanced programmed cell death at the highest dose of radiation (1.5 Gy). The total percentage (mid + late) of cells undergoing apoptosis at this dose was observed to be 33%.

### 3.3. Annexin V Detection

**Figure 3:**
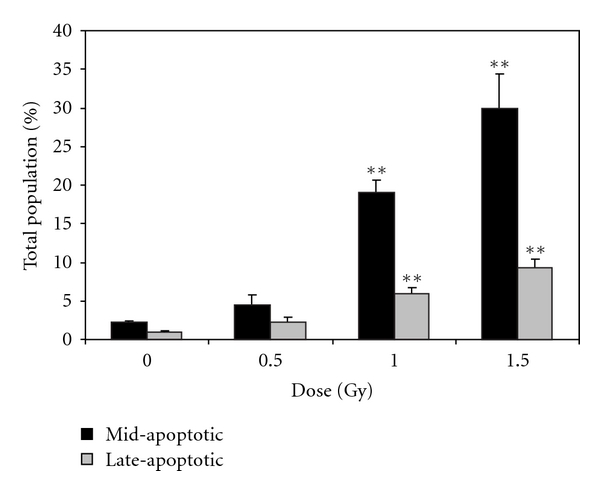
The percentage of mid- and late-apoptotic cells as a function of dose, 4 days after exposure to *α*-radiation as indicated by the annexin assay. Results are based on *n* = 3 biological replicates. **indicates statistically significant differences compared to the nonirradiated control (*P* ≤ .01). Figure is plotted as means ± SEM.

In addition to assessing apoptosis using the multicaspase detection assay, annexin V was also used as an alternative marker for cell death. Statistically significant differences in Annexin V^+^/PI^−^ and Annexin V^+^/PI^+^ (indicating mid-apoptosis and late-apoptosis, resp.), were observed at the medium and high (1.0 and 1.5 Gy) doses of *α*-particle irradiation (Figure 3). Mid-apoptotic cell population percentages in exposed samples ranged from approximately 5–30%, with late-apoptotic populations lying between 2–10%. It is interesting to note that while comparable trends were observed using the two apoptosis detection assays and total percentage of apoptotic cells were similar, the multicaspase assay appeared to detect earlier stages of apoptosis more efficienty than did the Annexin V assay, as evidenced by the relative percentages of mid- versus late-stage apoptosis detected by these two techniques. This may be attributed to the sensitivity in detection capability of these two markers for the intrinsic and extrinsic apoptotic pathways.

### 3.4. Gene Expression

THP-1 cells were exposed to three doses of *α*-particle radiation, and a time course of the apoptotic response was monitored over 7 days (data not shown) using annexin V detection. A strong response with a high percentage of apoptotic cells was observed at 4 days postexposure. At this time point, eighty-four genes were screened for differential expression following *α*-particle radiation treatment (Table 1). Among these 84 genes, a total of 27 genes were shown to be statistically significant (*P* ≤ .05) relative to the non-irradiated control samples. This included 15 genes which responded to all three doses tested (Table 2(a)), 7 genes which responded to the medium and high dose (Table 2(b)), and 5 genes which were found to be significant only at the highest dose tested (Table 2(c)). Of the 15 dose-responsive genes, one third was upregulated and two thirds were downregulated. Highly modulated genes included TNF-*α*, CASP5, and TNFRSF9 with fold changes (FC) of 4.25, 2.93, and 2.87, respectively, after exposure to 1.5 Gy of *α*-particle radiation. Approximately 30% of the genes responding to the medium and high dose were upregulated while 70% were downregulated. Of the genes which responded at only the medium and high dose, only two displayed ∣FC∣  ≥  2.00. These genes were BCL2A1 and CD40LG with FCs of 2.03 and −2.56, respectively. Of the genes which responded only to the highest dose of *α*-particle radiation, 40% were upregulated and 60% were downregulated, with two genes showing ∣FC∣  ≥  1.5 after the highest dose. These genes were TRAF4 (FC = 1.94) and BCL2L2 (FC = −1.54). Overall, the majority of genes tended to be downregulated.

### 3.5. PTX Suppression of TNF-*α* Expression

**Figure 4:**
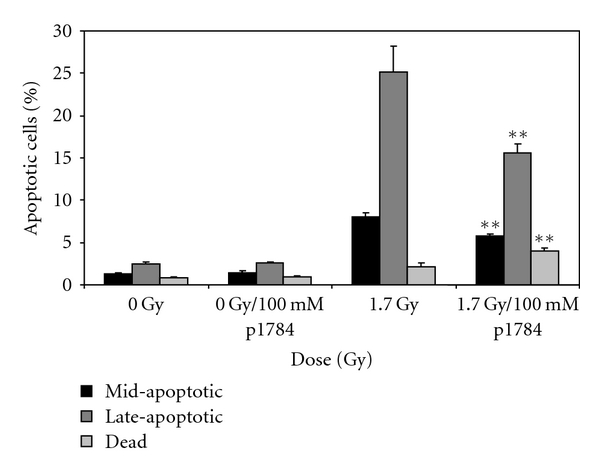
Pentoxifylline suppression of TNF-*α* expression. THP-1 cells were exposed to 1.7 Gy of *α*-particle radiation in the presence of 100 mM pentoxifylline. Apoptosis was determined using the Guava caspase reagent. Mean values ± SEM are shown. **represents *P* < .01 relative to 1.7 Gy treatment group, *n* = 6 biological replicates.

In order to confirm the involvement of TNF-*α* in cell apoptotic initiation, cells were incubated with PTX, a known inhibitor of TNF-*α* mRNA expression. The addition of 100 mM of this inhibitor resulted in statistically significant decrease (*P* ≤ .01) in the percentage of mid- to late-apoptotic cells (Figure 4) as measured by the multicaspase assay. The total percentage of (mid- and late-) apoptotic cells in samples treated with PTX following exposure to 1.7 Gy of *α*-particle radiation was ~21%. Therefore, on average, the percent of apoptotic cells decreased by 12% in the presence of PTX.

### 3.6. X-Irradiation and PTX Suppression of TNF-*α* and Associated Transcript Changes

**Figure 5:**
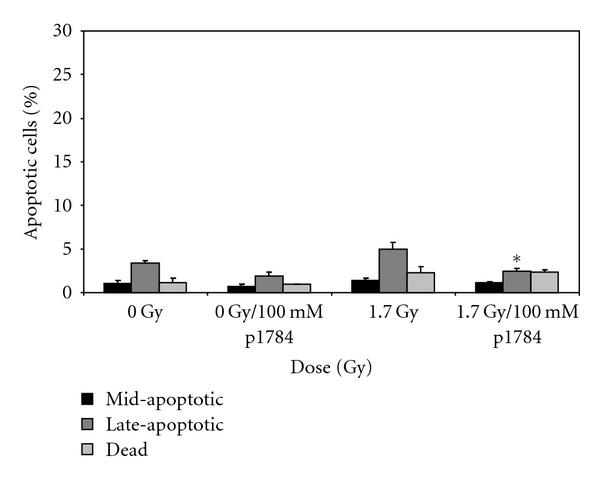
The pentoxifylline suppression of TNF-*α* expression. THP-1 cells were exposed to 1.7 Gy of X-radiation in the presence of 100 mM pentoxifylline (P1784-PTX). Apoptosis was determined using the Guava caspase reagent. Mean values ± SEM are shown. *represent *P* <.05, *n* = 6 biological replicates.

THP-1 cells that were exposed to equivalent doses of X-rays showed an attenuated apoptotic response relative to cells exposed to *α*-particle radiation. At the highest dose of irradiation (1.7 Gy, Figure 5) a total (mid- and late-), of 7% of the cells were shown to be apoptotic. An assessment of gene expression changes at three doses of radiation showed the expression of six statistically significant genes. Expression levels were shown to be on average <1.5 fold higher relative to the control samples (Table 3). Among the six genes, TNF-*α* and Fas were found to be common to the *α*-irradiated cells. Incubation of the cells with PTX resulted in statistically significant decrease (*P* ≤ .05) in the percentage of late-apoptotic cells (Figure 5) as measured by the multicaspase assay.

## 4. Discussion

In this paper, apoptosis was analyzed in a human monocyte-derived cell-line (THP-1) exposed to *α*-particle radiation (high LET) and X-rays (low LET). A number of transcripts associated with the apoptotic process were examined for differential expression. In addition, key targets of the apoptotic cascade including caspase enzyme activity and phosphatidylserine expression on the outer leaflet of the plasma membrane of THP-1 cells were monitored following exposure to the two radiation types. The purpose was to obtain a profile of genes associated with a high-LET radiation stressor, *α*-particle radiation and contrast it to a low-LET radiation stressor under similar dose rate conditions. This would allow for a better assessment of the biological impact which may in turn provide insight into the carcinogenic properties associated with *α*-particle radiation exposure.

To assess the apoptotic response following radiation exposure, the levels of two apoptotic markers (caspase enzyme activity and annexin V binding of externalized phosphatidylserine) were examined. Caspases play a central role in the apoptotic cell death process as they are involved in the break down of structural components of the cytoskeleton and activate cytokines [21]. By using a multicaspase detection analysis, the percentage of cells in various stages of apoptosis was determined following *α*-particle irradiation and X-irradiation at a low dose rate of exposure. Cells that were exposed to *α*-particle radiation were considerably more damaged and apoptotic four days postexposure relative to X-irradiated samples. For cells exposed to *α*-particle radiation, approximately 33% of the total cell population was apoptotic at the higher doses of irradiation when compared to the untreated control samples. Similar results were obtained when using annexin V as an alternative marker for detecting apoptosis. However, a comparison of the response for X-irradiation showed that only 5% of the cells were apoptotic relative to the control cells at the highest dose. To determine if the differential apoptotic response was attributed to gene modulations, the expression of 84 genes involved in the cell death pathway was examined.

Among the 84 genes screened, 15 genes were obtained that were differentially expressed at all three doses of *α*-particle radiation exposure, 7 genes were expressed at the high (1.5 Gy) and medium (1.0 Gy) doses and only 5 genes were expressed at the highest dose (1.5 Gy). In contrast, only 6 genes were observed to be modulated at the high (1.5 Gy) and medium (1.0 Gy) doses, using X-rays. Among these genes, TNF-*α* and Fas were shown to be common to the two radiation types. However, expression levels of TNF-*α* were markedly higher in *α*-particle exposed samples. TNF-*α* displayed expression levels four times those of the control nonirradiated cells in *α*-treated cells whereas only a 1.5-fold increase in TNF-*α* expression levels was observed in X-irradiated cells. TNF-*α* is a proinflammatory cytokine whose role is established in the pathogenesis of chronic inflammatory diseases [22]. TNF-*α* signaling leading to apoptosis is often mediated through the death domains of the receptor [23]. The death domain binds to an intracellular signaling moiety TNF-receptor-associated death domain protein (TRADD) [24]. This process can either lead to apoptosis via the caspases or through the TRAF molecules [22]. In this study, the observed upregulation of key genes associated with the TNF-*α* pathway including TRAF4, TNFRS9, and caspases would suggest one mode of apoptosis activation in THP-1 cells may be through the TNF-*α* receptor-based pathway [22]. 

To further confirm the involvement of TNF-*α* in radiation-induced apoptosis, a comparison was conducted in cells that were exposed to *α*-particle radiation and X-ray following treatment with PTX. The influence of PTX on THP-1 cells is as a suppressor of TNF-*α* mRNA expression and synthesis [25]. In samples exposed to *α*-particle radiation in the presence of PTX, there was a decrease in apoptotic cells of 12% relative to non-PTX-treated cells. In contrast to *α*-particle radiation, the pretreatment of cells with PTX resulted in a slight statistically significant (2%) decrease in apoptosis in X-ray treated cells. Despite the decrease in mid- and late-apoptosis with PTX suppression, it is interesting to note that a minor (~3%) increase is observed in the percentage of dead cells. This may be attributed to the activation of alternate apoptotic pathways, that is, through Fas initiation or necrotic cell death. In fact, in this study, both *α*- and X-irradiated samples displayed a ~2-fold induction in Fas expression. Fas is known to cause oligomerization of its receptor upon binding and is associated with the clustering of the death domains and binding of cofactor FADD. The FADD protein binds via its DED (Death Effector Domain) motif to a homologous motif in procaspas-8. Active caspase 8 then activates downstream caspases (Caspase 3, 5, and 7), committing the cell to apoptosis [26]. This process shares common intermediaries and mediators with the TNF-*α* signaling cascade [22]. Taken together, both TNF-*α* and/or Fas signaling cascades may be involved in radiation associated apoptosis.

Although apoptotic induction was seen with both radiation types, the X-ray response at the transcriptional and functional level was much less pronounced in comparison to *α*-particle radiation response, and this may in part be due to the initiation of repair mechanisms occurring at a low dose rate of exposure. Studies have shown that the relatively higher amount of clustered DNA lesions caused by *α*-particle radiation is potentially more difficult to repair than low-LET radiation (X-rays) and is more susceptible to mutagenic changes [27]. In a study conducted by Pinto et al. [28], primary human fibroblasts were irradiated with X-rays or *α*-particles, and the DNA double-strand breaks were resolved using pulsed-field gel electrophoresis. In X-irradiated samples, the majority of the DSBs were removed 24 post-irradiation. In contrast, for *α*-particles, approximately 85% of the DSBs remained after 24 h incubation. Thomas et al. [29] reported that ^210^Po *α*-particle radiation was 7 to 14 times more effective than X-rays in causing lethality in irradiated bovine aortic endothelial cells. In this study, X-irradiated samples may initiate repair mechanisms which would result in less-damaged cells and therefore decreased number of cells destined for apoptosis. Although not within the scope of this study, it would be interesting to further examine repair response genes in both *α*- and X-irradiated samples.

Among the 15 genes that were statistically expressed at all three doses of *α*-particle exposure, the majority were shown to be significantly downregulated and potentially unique to this radiation type as they were not induced in X-irradiated cells under the conditions of this study. Interestingly, a number of these *α*-particle dose-responsive genes have been shown to be associated anti-apoptotic functions and carcinogenesis. Type I insulin-like growth factor receptor (IGF1R) was among the genes with the highest fold change (FC = −2.2). Studies have shown that the activation of IGF1R promotes proliferation and inhibits apoptosis in a variety of cell types [30]. Mounting evidence has also demonstrated a crucial role of IGF1R signaling in the development and progression of cancer [31]. TNFRSF1A was also significantly downregulated by −2.1 fold in this study. This gene encodes for a protein which is known to activate NF-*κ*b and promote anti-apoptotic events [12]. Other genes which were downregulated in this study have also been shown to have anti-apoptotic roles; examples include BCLAF1, BCL2, and BIRC2. BIRC2 encodes a protein that inhibits apoptosis by binding to tumor necrosis factor receptor-associated factors TRAF1 and TRAF2, and by interfering with activation of ICE-like proteases [32]. BCLAF1 encodes a transcriptional repressor that interacts with several members of the BCL2 family of proteins. The overexpression of this protein induces apoptosis, which can be suppressed by coexpression of BCL2 proteins. The BCL2 family of proteins can induce or inhibit the release of cytochrome c into the cytosol through pro- or anti-apoptotic members respectively. Once cytochrome c enters the cytosol, it can activate caspase 9 and caspase 3, leading to apoptosis [32]. A similar downregulation in BCL2 expression was also observed in a study which exposed cells to ^222^Rn gas. This study showed that the exposure of MCF-7 cells to low dose rates of ^222^Rn induces underexpression of both BAX and BCL2 genes [33]. Overall, under the exposure conditions employed in this study, transcript modulations specific to the *α*-particle irradiated cells have been identified which may prove to be unique responses. However, further validation studies are required to confirm this, using different cell types, exposure conditions, and radiation types. 

In summary, evidence has been presented for increased levels of apoptosis in human monocytic cells exposed to *α*-particle radiation. Increases in apoptosis as determined by multicaspase and annexin V detections were directly correlated to the modulation in expression of a number of key apoptotic genes including caspases, BCL2, TNF-*α*, and Fas. This study further confirmed the highly damaging power of *α*-particle relative to X-rays and identified some unique *α*-particle responding genes with known carcinogenic functions. Further studies are needed to determine the interactions of other cellular responses associated with the expression of these apoptotic genes, as a number of these apoptotic genes have been shown to play roles in the carcinogenic pathways [16]. Future studies will further examine in depth the possible link between gene modulations and carcinogenesis following *α*-particle exposure using genome wide transcriptional profiling.

## Figures and Tables

**Table 1:** Functional gene groupings.

Functional families	Gene symbol
TNF ligand	*CD40LG, FASLG, LTA, TNF, TNFSF10, CD70, TNFSF8*
TNF receptor	*CD40, FAS, LTBR, TNFRSF10A, TNFRSF10B, TNFRS11B, TNFRSF1A, TNFRSF1B, TNFRSF21, TNFRSF25, CD27, TNFRSF9*
BCL-2	*BAD, BAG1, BAG3, BAK4, BAX, BCL2, BCL2A1, BCL2L1, BCl2L10, BCL2L11, BCL2L2, BCLAF1, BID, BIK, BNIP1, BNIP2, BNIP3, BNIP3L, HRK, MCL1 *
Caspase	*CASP1, CASP10, CASP14, CASP2, CASP3, CASP4, CASP5, CASP6, CASP7, CASP8, CASP9, *
IAP	*NAIP, BIRC2, BIRC3, BIRC4, BIRC6, BIRC8*
TRAF	*TRAF2, TRAF3, TRAF4*
CARD	*APAF1, BCL10, BIRC2, BIRC3, NOD1, CARD6, CARD8, CASP1, CASP2, CASP4, CASP5, CASP9*
Death domain	*CRADD, DAPK1, FADD, FAS, TNFRSF10A, TNFRSF10B, TNFRSF11B, TNFRSF1A, TNFRSF21, TNFRSF25, TRADD*
Death effector domain	*CASP8, CASP10, CFLAR, FADD*
CIDE domain	*CIDEA, CIDEB, DFFA*
P53 and DNA damage response	*ABL1, AKT1, APAF1, BAD, BAX, BCL2, BCL2L1, BID, CASP3, CASP6, CASP7, CASP9, GADD45A, TP53, TP53BP2, TP73*
Anti-apoptosis	*AKT1, BAG1, BAG3, BCL2, BCL2A1, BCl2L1, BCl2L10, BCL2L2L, BFAR, NAIP, BIRC2, BIRC3, BIRC4, BIRC6, BIRC8, BNIP1, BNIP2, BNIP3, BRAF, CASP2, CFLAR, FAS, IGF1R, MCL1, TNF, CDC27*

**Table:** (a)

Accession #	Gene name	0.5 Gy		1.0 Gy		1.5 Gy	
		FC	*P* Value	FC	*P* Value	FC	*P* Value
NM_000594	TNF	4.68	.00	5.43	.00	4.25	.00
NM_004347	CASP5	1.65	.04	2.78	.01	2.93	.00
NM_001561	TNFRSF9	2.82	.00	2.65	.00	2.87	.00
NM_000043	FAS	1.82	.00	1.75	.00	1.77	.01
NM_021960	MCL1	1.54	.00	1.59	.02	1.25	.02
NM_003879	CFLAR	−1.27	.02	−1.51	.01	−1.33	.01
NM_004323	BAG1	−1.21	.01	−1.41	.00	−1.39	.00
NM_001166	BIRC2	−1.49	.04	−1.44	.04	−1.46	.02
NM_012423	RPL13A	−1.30	.04	−1.38	.03	−1.64	.00
NM_014739	BCLAF1	−1.49	.01	−1.90	.00	−1.68	.00
NM_003805	CRADD	−1.36	.02	−1.51	.00	−1.72	.00
NM_001924	GADD45A	−1.79	.03	−1.90	.03	−1.72	.01
NM_000633	BCL2	−1.59	.00	−2.13	.00	−2.07	.00
NM_001065	TNFRSF1A	−2.31	.02	−2.51	.03	−2.11	.03
NM_000875	IGF1R	−1.92	.01	−1.77	.01	−2.21	.00

**Table:** (b)

Accession #	Gene name	1.0 Gy		1.5 Gy	
		FC	*P* Value	FC	*P* Value

NM_004049	BCL2A1	1.87	.00	2.03	.00
NM_004048	B2M	1.27	.02	1.34	.00
NM_001196	BID	−1.31	.01	−1.39	.00
NM_001229	CASP9	−1.38	.01	−1.39	.04
NM_000546	TP53	−1.38	.02	−1.43	.01
NM_014959	CARD8	−1.69	.01	−1.72	.00
NM_000074	CD40LG	−2.82	.03	−2.54	.03

**Table:** (c)

Accession #	Gene name	1.5 Gy	
		FC	*P* value

NM_004295	TRAF4	1.94	.02
NM_004281	BAG3	1.28	.02
NM_003842	TNFRSF10B	−1.24	.02
NM_003921	BCL10	−1.30	.04
NM_004050	BCL2L2	−1.53	.00

**Table 3:** Genes responsive at medium and high doses following X-rays. Statistically significant differentially expressed genes with corresponding *P* values and fold changes (FC) which were found to respond at 1.0 Gy and 1.5 Gy of X-radiation and were harvested 4 days after exposure listed by descending FC. Statistical cut-off at *P* ≤ .05, with *n* = 3 biological replicates.

Accession #	Gene name	1.0 Gy		1.5 Gy	
		FC	*P* value	FC	*P* value
NM_033341	BIRC8	1.54	.03	1.42	.05
NM_000043	FAS	1.58	.04	1.60	.03
NM_000639	FASLG	1.54	.03	1.42	.06
NM_000594	TNF	1.54	.00	1.52	.00
NM_002546	TNFRSF11B	1.54	.03	1.43	.06
NM_001252	CD70	1.44	.00	1.37	.03

## Abbreviations

^241^Am: Americium
^222^Rn: Radon
^226^Ra: Radium
MD: Mylar based plastic dishes
FBS: Fetal bovine serum
TBS: Triphosphate buffered saline
TST: TBS serum triton
PI: Propidium iodide
PBS: Phosphate buffered saline
FSC: Forward scatter
SSC: Side scatter
FITC: Fluorescein isothiocyanate
*α*: Alpha
MD: Mylar dish
RPMI: Royal park medical institute
ANOVA: Analysis of variance
qRTPCR: Quantitative real-time polymerase chain reaction
Ct: Comparitive threshold
IGF1R: Type I insulin-like growth factor receptor
TNF: Tumour necrosis factor *α*
FC: Fold change
TRADD: TNF receptor associated Death domain protein
PTX: Pentoxifylline.
